# Association Between mHealth Literacy and Hypertension-Related KAP Among Older Adults with Hypertension: Chain-Mediating Roles of Health Empowerment and Patient Activation

**DOI:** 10.3390/healthcare14142115

**Published:** 2026-07-14

**Authors:** Ying Han, Xinbao Lin, Jinfeng Xia, Ke Wang, Yaning Zhao, Fengmei Xing

**Affiliations:** 1 School of Public Health, North China University of Science and Technology, Tangshan 063000, China; hanying0423@ncst.edu.cn (Y.H.);; 2 College of Nursing and Rehabilitation, North China University of Science and Technology, Tangshan 063000, China

**Keywords:** hypertension, mHealth literacy, health empowerment, patient activation, hypertension-related KAP

## Abstract

**Highlights:**

**What are the main findings?**

- mHealth literacy was positively associated with hypertension-related knowledge, attitudes, and practices (KAP) among older adults with hypertension after adjusting for sociodemographic covariates.
- Health empowerment and patient activation were involved in statistically significant indirect associations, with the largest indirect association observed through health empowerment.

**What are the implications of the main findings?**

- Hypertension-related KAP in older adults may be related not only to mHealth literacy, but also to psychosocial self-management resources, such as perceived control and active engagement.
- Future community-based health education programs may consider integrating mHealth literacy support, empowerment-oriented education, and patient activation strategies.

**Abstract:**

Background: As mobile health (mHealth) technologies become increasingly integrated into chronic disease management, understanding how mHealth literacy is associated with hypertension-related knowledge, attitudes, and practices (KAPs) is important. However, the psychosocial factors involved in this association remain unclear. This study aimed to examine the association between mHealth literacy and hypertension-related KAP among older adults with hypertension and to explore whether health empowerment and patient activation were involved in statistically significant indirect pathways. Methods: A cross-sectional survey was conducted among 1500 community-dwelling older adults with hypertension in Hebei Province, China, between December 2024 and June 2025. Data were collected using the Problem-Based mHealth Literacy Scale, the Health Empowerment Scale for Elderly Patients with Chronic Disease, the Patient Activation Measure, and the Community Elderly Hypertension Patients Health Science Popularization Cognitive Scale. Partial correlation analyses were performed after controlling for sociodemographic covariates. An adjusted structural equation model was used to examine the hypothesized indirect pathway structure, and indirect effects were tested using bootstrapping with 5000 resamples. Results: mHealth literacy was positively associated with hypertension-related KAP. The total effect of mHealth literacy on hypertension-related KAP was 0.399 (95% CI: 0.347–0.499), including a direct effect of 0.290 (95% CI: 0.238–0.341) and a total indirect effect of 0.108 (95% CI: 0.082–0.137). The indirect pathway through health empowerment was statistically significant and accounted for the largest proportion of the total effect (indirect effect = 0.079, 95% CI: 0.059–0.103). The indirect pathway through patient activation was also statistically significant (indirect effect = 0.019, 95% CI: 0.003–0.035), as was the sequential indirect pathway through health empowerment and patient activation (indirect effect = 0.010, 95% CI: 0.006–0.016). Conclusions: In this cross-sectional sample of older adults with hypertension, mHealth literacy was positively associated with hypertension-related KAP. The data were consistent with indirect associations involving health empowerment and patient activation, with the largest indirect association observed through health empowerment. These findings suggest that mHealth literacy and psychosocial self-management resources may both be relevant to hypertension-related KAP. Further longitudinal or intervention studies are needed to clarify the temporal and causal nature of these associations.

## 1. Introduction

Hypertension is one of the most prevalent chronic non-communicable diseases worldwide and remains a major public health challenge, particularly among older adults, because of its association with cardiovascular morbidity, functional decline, and premature mortality [1,2]. In China, the prevalence of hypertension has reached 31.6%, whereas the control rate remains only 12.9% [3], below the global average of 23% [4]. Effective long-term management for older adults with hypertension depends not only on clinical treatment but also on the ability to understand disease-related information, form appropriate attitudes toward prevention and control, and engage in sustained self-management practices [5]. Therefore, improving hypertension-related knowledge, attitudes, and practices is an important component of community-based hypertension management.

Health education is widely recognized as an important strategy for improving disease-related knowledge and promoting healthier self-management behaviors [6]. However, mere exposure to health information does not necessarily translate into sustained behavioral change. The knowledge–attitudes–practices (KAP) framework provides a useful basis for examining how individuals acquire health knowledge, develop attitudes toward disease management, and translate these attitudes into daily practices [7,8]. In this study, hypertension-related KAP refers specifically to knowledge about hypertension prevention and control, attitudes toward long-term disease management, and self-management practices such as medication adherence, lifestyle regulation, blood pressure monitoring, and appropriate use of health services [9]. This disease-specific construct is distinct from general scientific literacy. Accordingly, the present study uses the term hypertension-related KAP to describe the primary outcome.

With the increasing integration of digital technologies into chronic disease management, mHealth has become an important avenue for older adults to access health information and self-management support [10]. mHealth literacy refers to an individual’s ability to access, understand, evaluate, and use health information and services delivered through mobile technologies [11]. Although previous studies have reported associations between mHealth literacy and treatment adherence, self-management, and chronic disease outcomes [12,13], most investigations have focused on direct associations [14,15]. Less is known about the psychosocial pathways through which mHealth literacy may be associated with hypertension-related KAP, especially among community-dwelling older adults.

Health empowerment and patient activation are two conceptually distinct psychosocial constructs that may be involved in indirect associations between mHealth literacy and hypertension-related KAP [16,17]. Health empowerment refers to perceived control, autonomy, and agency in health-related decision-making [18,19]. It emphasizes whether individuals feel capable of understanding their health condition, obtaining support, and participating in treatment and self-management decisions [20,21]. Patient activation, by contrast, refers to the knowledge, skills, confidence and readiness required to actively engage in health management [22]. Health empowerment mainly reflects perceived control and agency, whereas patient activation reflects preparedness and confidence for self-management action. Both constructs have been linked to self-management and health-related outcomes in chronic disease populations [23,24,25]. However, empirical evidence examining whether these constructs form indirect associations linking mHealth literacy to hypertension-related KAP remains limited.

**Figure 1:**
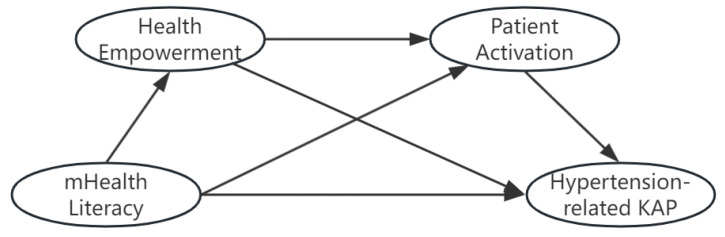
Diagram of the hypothetical model.

Based on this consideration, this study aimed to examine the association between mHealth literacy and hypertension-related KAP among older adults with hypertension and to explore whether health empowerment and patient activation are involved in this relationship. We hypothesized that mHealth literacy would be positively associated with hypertension-related KAP and that the data would be consistent with indirect pathways involving health empowerment and patient activation, including a sequential indirect association from health empowerment to patient activation (Figure 1). Given the cross-sectional design, these hypothesized pathways are interpreted as associative rather than causal. The findings may inform the development of future community-based digital health education and self-management support strategies for older adults with hypertension.

## 2. Materials and Methods

### 2.1. Study Design, Participants and Sample Size

This study employed a cross-sectional design. Data were collected between December 2024 and June 2025 from 1500 community-dwelling older adults with hypertension in three cities of Hebei Province, China (Shijiazhuang, Tangshan, and Zhangjiakou), selected to maximize socioeconomic diversity. Participants were recruited using convenience sampling from community settings.

Inclusion criteria were: (1) aged ≥60 years; (2) physician-diagnosed hypertension according to the 2023 Chinese Guidelines for the Management of Hypertension; (3) had resided in the survey area for at least 6 months; and (4) voluntary participation. Exclusion criteria included: (1) severe physical illness or psychiatric illness; (2) major hearing or language impairments preventing communication; or (3) secondary hypertension.

Sample size estimation followed Kendall’s guideline recommending 10–20 times the number of variables, with an additional 20% allowance for potentially invalid responses [26]. This study included 25 variables in total, comprising 8 demographic variables, 8 dimensions of the Problem-Based mHealth Literacy Scale (PB-mHLS), 5 dimensions of the Health Empowerment Scale for Elderly Chronic Disease Patients, one dimension of the Patient Activation Measure (PAM), and 3 dimensions of the Elderly Hypertension Patients Health Science Popularization Cognitive Scale. Based on this principle, the estimated required sample size ranged from 300 to 600. In addition, previous methodological literature suggested that a minimum sample size of 200 is generally desirable for SEM [27]. The final sample included 1500 participants, exceeding both requirements.

### 2.2. Measurements

#### 2.2.1. Sociodemographic Characteristics

A self-administered general information questionnaire was developed by the research team. It collected age, gender, education, marital status, monthly income, occupational category, living arrangement, and duration of hypertension.

#### 2.2.2. Problem-Based mHealth Literacy Scale (PB-mHLS)

mHealth literacy was measured using the PB-mHLS developed by Zhang and Li to measure individuals’ ability to identify, access, understand, evaluate, communicate, and apply health information and services in mobile health contexts [28]. The scale comprises 33 items across eight dimensions: desire for mobile health, mobile device operational skills, access to mobile health information, access to mobile health services, understanding medical terminology, mobile-device-based patient-provider communication, evaluation of mobile health information, and mobile health decision-making. Each item is scored on a 5-point Likert scale from 1 (“strongly disagree”) to 5 (“strongly agree”). Total scores range from 33 to 165, with higher scores indicating greater mHealth literacy. In this study, the scale demonstrated excellent internal consistency, with a Cronbach’s α of 0.950.

#### 2.2.3. Health Empowerment Scale for Elderly Chronic Disease Patients

Health empowerment was assessed using the Health Empowerment Scale for Elderly Chronic Disease Patients developed by Yang [29] to assess the extent to which older patients perceive themselves as capable of participating in disease management, obtaining health-related support, taking responsibility for health decisions, and reconstructing self-management confidence. The scale consists of 26 items grouped into five dimensions: responsibility belief, self-reconstruction, knowledge acquisition, treatment participation, and support acquisition. Each item is rated on a 5-point Likert scale from 1 (“strongly disagree”) to 5 (“strongly agree”). Total scores range from 26 to 130, with higher scores indicating greater health empowerment. In this study, the scale demonstrated high internal consistency, with a Cronbach’s α of 0.942.

#### 2.2.4. Patient Activation Measure (PAM)

Patient activation was assessed using the 13-item PAM [30]. The original PAM was developed using Rasch psychometric methods and conceptualized activation as a developmental construct involving progressively greater patient participation in health management. Subsequent work developed and tested the 13-item short form, which retained the unidimensional structure and interval-level measurement properties of the original measure while reducing respondent burden. Each item assesses patient knowledge, skills, and confidence in self-management. Items are rated on a 5-point Likert scale from 0 (“not applicable”) to 4 (“strongly agree”), with 1 representing “strongly disagree”. According to the standard scoring procedure, raw scores were transformed into standardized scores ranging from 0 to 100. Higher scores indicate greater patient activation. In this study, the scale demonstrated excellent internal consistency, with a Cronbach’s α of 0.945.

#### 2.2.5. Community Elderly Hypertension Patients Health Science Popularization Cognitive Scale

Hypertension-related KAP was measured using the Community Elderly Hypertension Patients Health Science Popularization Cognitive Scale, developed by Zhang based on the knowledge–attitude–practice framework for community-dwelling older adults with hypertension [31]. The scale was designed to assess disease-specific health science popularization cognition in this population, including understanding of hypertension-related health knowledge, attitudes toward disease prevention and control, and engagement in self-management practices. Because the construct assessed by this scale is disease-specific rather than general scientific literacy, it is described in the present study as hypertension-related KAP. The scale comprises 38 items across three dimensions: knowledge, attitude, and practice. Each item is rated on a 5-point Likert scale from 1 (“strongly disagree”) to 5 (“strongly agree”). Total scores range from 38 to 190, with higher scores indicating better hypertension-related KAP. In this study, the scale showed excellent internal consistency, with a Cronbach’s α of 0.975.

### 2.3. Data Collection and Quality Control

Data were collected using an online questionnaire hosted on the Wenjuanxing platform (https://www.wjx.cn), a widely used online survey tool in China. Before beginning the survey, participants were informed about the study purpose, procedures, confidentiality, and voluntary nature of participation. Electronic informed consent was obtained from all participants before data collection.

Although the questionnaire was designed to be self-administered, some participants required assistance because of advanced age, visual difficulty, or difficulty completing the online questionnaire independently. For these participants, trained investigators read the questionnaire items aloud when necessary and recorded the participants’ responses verbatim. Investigators were instructed not to explain item meanings beyond standardized wording, provide examples, suggest answers, or guide response selection.

Several measures were used to ensure data quality: (1) the platform was configured to allow only one response per IP address; (2) all questionnaire items were set as mandatory; and (3) questionnaires were screened after submission. Responses were excluded if they met any of the following criteria: (1) under 2 min; (2) obvious logical inconsistencies in responses; or (3) patterned responses exceeding 80% of items. A total of 1673 questionnaires were collected, of which 173 were excluded. Thus, 1500 responses were retained, yielding an effective response rate of 89.66%.

### 2.4. Statistical Analysis

All analyses were conducted using SPSS 27.0 software and AMOS 25.0. Continuous variables were expressed using mean ± standard deviation (SD), and categorical variables as frequencies and percentages. Group differences in the main study variables across participant characteristics were examined using independent-samples t-tests, one-way ANOVA, as appropriate. In preliminary analyses, sociodemographic variables associated with the main study variables were treated as potential covariates. Partial correlation analyses were then performed to examine the associations among key variables after adjusting covariates.

A structural equation model (SEM) was conducted using AMOS 25.0 to examine the proposed indirect pathway model. Significant sociodemographic variables were included as observed covariates in the adjusted model. Model fit was evaluated using χ^2^/df, NFI, GFI, RFI, CFI, TLI, IFI, and RMSEA. Indirect effects were tested using the bootstrap method, and 95% confidence intervals (CI) were calculated. An indirect effect was considered statistically significant if the 95% CI did not include zero. A two-sided *p*-value of <0.05 was considered statistically significant.

### 2.5. Ethical Considerations

This study was approved by the Ethics Committee of North China University of Science and Technology (approval number: 2024947). All procedures adhered to the Declaration of Helsinki and the Ethical Review Measures for Life Sciences and Medical Research Involving Humans. Before participation, all individuals were informed about the study and provided informed consent. Participants were informed that participation was voluntary and that they could withdraw from the study at any time without consequences.

## 3. Results

### 3.1. Sociodemographic Characteristics

A total of 1500 older adults with hypertension were included in the final analysis. The mean age was 72.46 ± 9.81 years (range: 60–86). Of the participants, 764 (50.9%) were men and 736 (49.1%) were women. Detailed characteristics and group comparisons are presented in Table 1.

### 3.2. Scores of the Main Variables and Group Comparisons

The overall hypertension-related KAP score was (120.05 ± 26.72). Scores for other main variables were: mHealth literacy (95.43 ± 19.66), health empowerment (81.59 ± 15.67), and patient activation (47.89 ± 11.18). Univariate analyses showed that several sociodemographic characteristics were significantly associated with one or more of the main study variables. Detailed results are presented in Table 1.

### 3.3. Common Method Bias Test

Harman’s single-factor test was conducted to assess common method bias. Unrotated principal component analysis showed that the first factor explained 30.85% of the total variance, below the commonly used 40% threshold [32].

### 3.4. Partial Correlation Analysis

Sociodemographic variables with *p* < 0.05 in univariate analyses were treated as potential covariates. The results showed that all main variables were significantly and positively correlated with one another. Detailed results are presented in Table 2.

### 3.5. SEM and Indirect Effect Analysis

**Figure 2:**
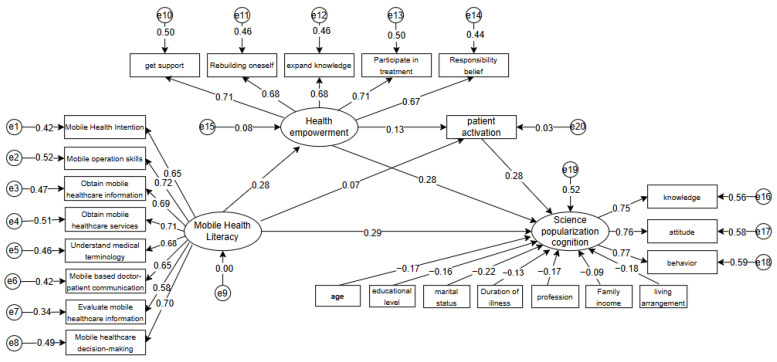
Adjusted SEM of the association. Note: e represents the residual-term (error-term) of the measurement model.

SEM was conducted to examine the hypothesized relationships among variables. Sociodemographic variables that were significantly associated with the main study variables in univariate analyses were included as covariates in the adjusted structural equation model. The adjusted model showed acceptable fit: χ^2^/df = 3.187, NFI = 0.923, GFI = 0.951, RFI = 0.914, CFI = 0.946, TLI = 0.939, IFI = 0.946, and RMSEA = 0.038. All indices met established criteria for SEM, indicating an acceptable model fit. mHealth literacy was positively associated with health empowerment, patient activation, and hypertension-related KAP. Health empowerment was positively associated with patient activation and hypertension-related KAP. Patient activation was positively associated with hypertension-related KAP. The corresponding path coefficients are presented in Figure 2.

Bootstrap analyses were conducted to examine the indirect effects in the structural model. The total effect of mHealth literacy on hypertension-related KAP was 0.399 (SE = 0.026, 95% CI: 0.347–0.499). The direct effect was 0.290 (SE = 0.026, 95% CI: 0.238–0.341), accounting for 72.68% of the total effect. The total indirect effect was 0.108 (SE = 0.014, 95% CI: 0.082–0.137), accounting for 27.07% of the total effect.

Three indirect pathways were statistically significant, see Table 3. Pathway 1, through health empowerment, had the largest indirect effect, with an effect estimate of 0.079 (SE = 0.011, 95% CI: 0.059–0.103), accounting for 19.80% of the total effect. Pathway 2, through patient activation, had an indirect effect of 0.019 (SE = 0.008, 95% CI: 0.003–0.035), accounting for 4.76% of the total effect. Pathway 3, through health empowerment and patient activation sequentially, had an indirect effect of 0.010 (SE = 0.003, 95% CI: 0.006–0.016), accounting for 2.51% of the total effect. Because none of the bootstrap confidence intervals included zero, all three indirect pathways were statistically significant.

## 4. Discussion

This study examined how mHealth literacy was associated with hypertension-related KAP among older adults with hypertension after adjustment for sociodemographic covariates. The adjusted structural model showed a positive association between mHealth literacy and hypertension-related KAP. Health empowerment and patient activation were also involved in this association, with the largest indirect association observed through health empowerment and smaller contributions from patient activation. These findings suggest that psychosocial self-management resources, particularly perceived control and active engagement, may be important when interpreting the relationship between mHealth literacy and hypertension-related KAP. However, given the cross-sectional design, these findings should be interpreted as adjusted associations rather than evidence of temporal or causal mediation.

In the present study, mHealth literacy was positively associated with hypertension-related KAP among older adults with hypertension. This finding is consistent with previous studies linking digital health literacy to health-related behaviors and chronic disease self-management [12,15,33,34]. In particular, Wu et al. [33] reported that eHealth literacy was associated with self-management among older Chinese patients with chronic non-communicable diseases. Older adults with higher mHealth literacy may be better able to obtain, understand and use hypertension-related information through mobile devices, and communicate more effectively with healthcare providers [35,36,37]. Such abilities are particularly important for long-term hypertension management, which often requires repeated access to clear and trustworthy information. Previous studies also suggest that digital health literacy is associated with more active engagement in chronic disease management [17,33,38], which may help explain the positive association between mHealth literacy and hypertension-related KAP observed in this study.

In the present study, health empowerment accounted for the largest indirect association between mHealth literacy and hypertension-related KAP. This finding suggests that the relationship between mHealth literacy and hypertension-related KAP may be closely related to older adults’ perceived control, agency, and confidence in hypertension self-management. Health empowerment reflects individuals’ perceived ability to obtain and use health resources, participate in health-related decisions, and manage chronic conditions in daily life [19,38,39]. This construct is closely connected with the attitude and practice components of hypertension-related KAP, because empowered individuals may be more likely to recognize the value of scientific disease management and implement recommended self-management behaviors [16,20,40]. Consistent with this interpretation, previous studies have linked empowerment to chronic disease self-management and health-related behaviors [18,21]. Therefore, health empowerment may represent a particularly relevant psychosocial component in the association between mHealth literacy and hypertension-related KAP among older adults with hypertension.

In the present study, patient activation was also involved in an indirect association between mHealth literacy and hypertension-related KAP, although its contribution was smaller than that of health empowerment. This finding suggests that active participation in health management may be a relevant, but less prominent, psychosocial component in this association. Patient activation reflects individuals’ knowledge, skills, confidence, and readiness to take an active role in managing their health [30,41]. Previous studies have linked higher patient activation to better self-management and health-related outcomes among people with chronic diseases [42,43]. Bonifanti et al. also reported that eHealth literacy was associated with patient activation after accounting for sociodemographic factors [44], which is relevant to the adjusted model in the present study. The relatively smaller indirect association through patient activation may reflect the specific challenges faced by older adults, including limited digital familiarity, lower confidence in using technology, and continued reliance on healthcare professionals [45].

The present study also supported a small sequential indirect association involving health empowerment and patient activation. This finding suggests that perceived control and active engagement may be interconnected psychosocial factors in the association between mHealth literacy and hypertension-related KAP. In this pattern, higher health empowerment was associated with higher patient activation, indicating that older adults who perceive greater control and confidence in managing hypertension may also report greater readiness to participate in self-management. This interpretation is consistent with previous research suggesting that empowerment and activation are related psychosocial resources in chronic disease management [20,38,46,47]. Green et al. [48] also highlighted that mediation-type psychosocial associations in older adults with chronic conditions should be interpreted cautiously and within their population context. Therefore, although the sequential indirect association was statistically supported, it should not be viewed as a confirmed causal chain. Reverse or reciprocal explanations are possible; for example, more activated individuals may be more likely to seek digital health information and report higher mHealth literacy [49,50].

These findings may have implications for community-based health education and self-management programs for older adults with hypertension. Such programs may consider combining mHealth literacy support, empowerment-oriented education, and patient activation strategies. First, mHealth literacy support could include hands-on guidance on using mobile devices to search for hypertension information, identify reliable sources, interpret blood pressure-related messages, and avoid misleading online content. Second, empowerment-oriented education could use scenario-based discussion to help older adults understand treatment recommendations, prepare questions for clinical visits, and participate in decisions about medication, diet, exercise, and blood pressure monitoring. Third, activation-oriented strategies could include simple self-monitoring plans, individualized goal setting, and follow-up feedback to encourage older adults to discuss management difficulties with healthcare professionals. Future longitudinal and intervention studies are needed to examine whether these integrated components can improve hypertension-related KAP.

### Limitations and Future Directions

Several limitations should be acknowledged when interpreting the findings. First, the cross-sectional design precludes conclusions about temporal ordering or causal relationships among mHealth literacy, health empowerment, patient activation, and hypertension-related KAP. Future longitudinal or intervention studies are needed to clarify the direction and causal nature of these associations. Second, participants were recruited using convenience sampling from three cities in one province, which may limit the generalizability of the findings and introduce selection bias. Future studies should include more representative samples from different geographic and socioeconomic settings. Third, the online questionnaire format may have underrepresented older adults with limited digital access or lower digital skills. Future research should consider mixed-mode data collection to include older adults with varying levels of digital access and skills. Fourth, although trained investigators assisted some participants using standardized procedures, assisted completion may have influenced responses, particularly for items related to mHealth literacy. Future studies should record the extent of assisted completion and examine its potential influence on measurement. Fifth, all main variables were measured using the same-source self-reported questionnaires in a single survey session. Although Harman’s single-factor test did not indicate a dominant single factor, this test cannot fully rule out common method variance. Recall bias, social desirability, and positive response tendencies may still have influenced the observed associations. Future studies should use multi-source data, objective indicators, or more robust procedural and statistical approaches to reduce and assess common method bias. Sixth, although the adjusted structural model included measured sociodemographic covariates, residual confounding from unmeasured factors, such as cognitive function, hypertension severity, medication burden, family support, healthcare access, and actual digital device use, cannot be ruled out. Future studies should incorporate these factors to further test the robustness of the observed associations.

## 5. Conclusions

In conclusion, mHealth literacy was positively associated with hypertension-related KAP among older adults with hypertension after adjusting for sociodemographic covariates. Health empowerment and patient activation were involved in indirect associations between mHealth literacy and hypertension-related KAP, with the largest indirect association observed for health empowerment and a smaller sequential association involving both health empowerment and patient activation. These findings suggest that mHealth literacy, perceived control, and active engagement in self-management may be relevant to hypertension-related KAP. Further longitudinal or intervention studies are needed to clarify the temporal and causal nature of these associations.

## Figures and Tables

**Table 1:** Comparison of main study variables across participant characteristics (mean ± SD, *n* = 1500).

Characteristics	Classification	*n* (%)	mHealth Literacy	Health Empowerment	Patient Activation	Hypertension-Related KAP
Gender	Male	764 (50.93)	95.35 ± 19.37	80.80 ± 15.84	47.55 ± 11.46	120.17 ± 26.58
	Female	736 (49.01)	95.35 ± 13.67	82.41 ± 15.46	48.25 ± 10.86	119.93 ± 26.88
	*t*		−0.162	−1.984	−1.216	0.169
	*P*		0.872	0.047	0.224	0.866
Age (year)	60–65	285 (19.00)	96.99 ± 20.56	83.90 ± 15.50	49.77 ± 11.37	129.53 ± 28.30
	66–70	257 (17.13)	95.71 ± 19.39	84.20 ± 15.89	48.65 ± 11.23	125.42 ± 27.41
	70–75	330 (22.00)	96.19 ± 19.06	81.82 ± 15.12	48.26 ± 10.83	122.25 ± 23.23
	76–80	328 (21.87)	93.41 ± 19.64	79.11 ± 15.73	47.45 ± 10.74	114.81 ± 25.67
	>80	300 (20.00)	95.08 ± 19.63	79.62 ± 15.55	45.54 ± 11.43	107.77 ± 24.66
	*F*		1.476	6.702	5.920	28.313
	*P*		0.207	0.000	0.000	0.000
Education	Elementary school	398 (26.53)	89.19 ± 18.34	78.60 ± 16.06	45.02 ± 11.08	103.27 ± 19.40
	Middle school	409 (27.27)	91.31 ± 18.31	78.77 ± 15.90	45.72 ± 11.57	106.81 ± 24.66
	High school	358 (23.87)	98.29 ± 19.40	83.43 ± 15.71	49.14 ± 11.09	127.67 ± 26.11
	College/Associate	335 (22.33)	101.45 ± 19.88	84.75 ± 14.17	50.99 ± 9.96	138.26 ± 18.52
	*F*		33.904	15.672	24.934	26.058
	*P*		0.000	0.000	0.000	0.000
Marital status	Married	451 (30.07)	103.95 ± 18.92	89.05 ± 14.08	52.79 ± 9.54	151.40 ± 12.20
	Divorced	412 (27.47)	94.97 ± 18.62	80.51 ± 14.43	48.26 ± 10.43	117.41 ± 12.97
	Single	230 (15.33)	94.42 ± 18.96	79.30 ± 14.42	46.86 ± 11.29	114.79 ± 16.07
	Widowed	407 (27.13)	87.03 ± 18.02	75.71 ± 16.05	42.69 ± 11.13	90.97 ± 13.87
	*F*		59.516	62.251	66.889	53.406
	*P*		0.000	0.000	0.000	0.000
Occupational category	Agricultural	294 (19.60)	97.63 ± 20.18	83.79 ± 15.33	48.93 ± 11.31	129.39 ± 27.10
	Industrial worker	246 (16.40)	97.49 ± 20.23	83.48 ± 15.14	48.96 ± 11.16	127.47 ± 28.41
	Unemployed	309 (20.60)	94.47 ± 19.57	81.03 ± 15.39	48.07 ± 10.61	118.42 ± 24.04
	Public institution employee	330 (22.00)	94.53 ± 18.97	80.57 ± 15.49	47.77 ± 10.88	118.04 ± 23.91
	Others	321 (21.40)	93.69 ± 19.33	79.72 ± 16.49	46.08 ± 11.71	109.46 ± 25.82
	*F*		2.586	3.975	3.361	29.138
	*P*		0.035	0.003	0.010	0.000
Monthly income/(yuan)	<3000	445 (29.67)	96.50 ± 19.37	81.80 ± 16.04	48.79 ± 10.89	123.64 ± 26.85
	3000–5000	544 (36.27)	96.03 ± 19.78	82.79 ± 15.19	48.43 ± 10.62	123.76 ± 24.93
	>5000	511 (34.07)	93.86 ± 19.74	80.14 ± 15.74	46.55 ± 11.87	112.98 ± 27.06
	*F*		2.556	3.864	5.800	28.150
	*P*		0.078	0.022	0.003	0.000
Living arrangement	With spouse	288 (19.20)	99.48 ± 19.99	85.23 ± 14.75	50.55 ± 11.19	133.03 ± 25.55
	With children	390 (26.00)	97.51 ± 18.55	83.10 ± 14.78	48.54 ± 10.45	126.81 ± 21.40
	Living with family	455 (30.33)	92.72 ± 19.92	79.13 ± 16.45	46.41 ± 11.34	112.08 ± 25.72
	Care facility	367 (24.67)	93.40 ± 19.53	80.18 ± 15.64	46.97 ± 11.31	122.57 ± 28.06
	*F*		9.877	11.353	9.544	60.520
	*P*		0.000	0.000	0.000	0.000
Duration of hypertension (year)	<5	375 (25.00)	97.24 ± 19.31	82.42 ± 16.04	49.04 ± 10.48	124.60 ± 28.95
	5–10	650 (43.33)	96.36 ± 19.94	82.25 ± 15.34	48.34 ± 11.50	121.28 ± 26.00
	>10	475 (31.67)	92.72 ± 19.32	80.03 ± 15.73	46.38 ± 11.12	114.78 ± 24.97
	*F*		6.893	3.458	6.909	15.681
	*P*		0.001	0.032	0.001	0.000

Note: SD, standard deviation.

**Table 2:** Partial correlation analysis among the main variables (*r*).

	1	2	3	4
1. mHealth literacy	1			
2. Health empowerment	0.324 **	1		
3. Patient activation	0.279 *	0.230 *	1	
4. Hypertension-related KAP	0.385 **	0.368 **	0.380 **	1

Note: * *p* < 0.05; ** *p* < 0.01; Partial correlations were adjusted for gender, age, educational level, marital status, occupation category, monthly income, living arrangement and duration of illness.

**Table 3:** Results of bootstrap analysis of indirect effects.

Effect	Pathway	Effect Estimate	Boot S.E.	LLCL	ULCL	Proportion (%)
Total effect		0.399	0.026	0.347	0.499	——
Direct effect	Direct pathway	0.290	0.026	0.238	0.341	72.68%
Total indirect effect		0.108	0.014	0.082	0.137	27.07%
Indirect effect	Pathway 1	0.079	0.011	0.059	0.103	19.80%
	Pathway 2	0.019	0.008	0.003	0.035	4.76%
	Pathway 3	0.010	0.003	0.006	0.016	2.51%

Note: S.E., standard error; CI, confidence interval. Indirect effects were considered statistically significant when the 95% bootstrap CI did not include zero. Proportion = indirect effect/total effect × 100%.

## Data Availability

The data that support the findings of this study are available from the corresponding author upon reasonable request due to privacy and ethical restrictions involving sensitive personal and health information of elderly participants.
